# Effects of *Trichoderma* Biostimulation on the Phenolic Profile of Extra-Virgin Olive Oil and Olive Oil By-Products

**DOI:** 10.3390/antiox9040284

**Published:** 2020-03-27

**Authors:** Irene Dini, Giulia Graziani, Francesca Luisa Fedele, Andrea Sicari, Francesco Vinale, Luigi Castaldo, Alberto Ritieni

**Affiliations:** 1 Department of Pharmacy, University of Naples Federico II, Via Domenico Montesano 49, 80141 Napoli, Italy; ritialb@unina.it; 2 LINFA SCARL. Via Zona Industriale Porto San Salvo, 89900 Vibo Valentia, Italy; ricerca@laboratoriolinfa.it (F.L.F.); andrea@laboratoriolinfa.it (A.S.); 3 Department of Veterinary Medicine and Animal Productions, University of Naples Federico II, Via Federico Delpino 1, 80137 Napoli, Italy; 4 Institute for Sustainable Plant Protection, National Research Council, Via Università 133, 80055 Portici (NA), Italy; 5 Department of Clinical Medicine and Surgery, University of Naples Federico II, Via S. Pansini 5, 80141 Napoli, Italy; luigi.castaldo2@unina.it

**Keywords:** *Trichoderma* spp., EVOO, olive leaves, harzianic acid, phenolic identification, HRMS-Orbitrap, antioxidant activity

## Abstract

Olive trees are grown on five continents. Fertilization of fields, pest control management, olive leaves, olive pomaces, and olive mill wastewaters have a substantial environmental impact. It is possible to reduce this problem by using organic products to cultivate and decrease olive oil processing waste by recovering the bioactive molecules. In this work, the effects of biostimulation, with beneficial microbes belonging to the *Trichoderma* genera, and with *Trichoderma* secondary metabolites (6PP and the HA) were evaluated on the phenolic profile and the antioxidant potential of extra-virgin olive oil (EVOO) and olive leaf samples to make them more commercially attractive as a source of phytochemicals useful for the pharmaceutical, cosmetic, and food industries. Phenolics were identified and quantified by a spectrometer method using Q Exactive Orbitrap UHPLC-MS/MS (Ultra High Pressure Liquid Chromatography). Antioxidant activity was evaluated spectrophotometrically by the DPPH test. The use of *Trichoderma strains*, 6PP (6-Pentyl-α-Pyrone) and HA (Harzianic Acid), was demonstrated as an effective strategy to increase the leaves’ economic value as a source of phytochemicals (flavonoids, lignans, and oleuropein) useful for food, pharmaceutical, and cosmetic industries.

## 1. Introduction

Olive production is a critical economic sector in many rural regions around the globe. The leading European producers are Spain (2.4 million ha), Italy (1.4 million ha), Greece (1 million ha), Portugal (0.5 million ha), and France (40,000 ha). Other cultivation countries are Tunisia, Turkey, Syria, Morocco, Algeria, Cyprus, Israel, Egypt, Libya, Lebanon, Jordan, Argentina, Mexico, Chile, Peru, Australia, USA, and China [1]. A new eco-friendly way to promote plant development and raise crop productivity is biostimulation with microbes. Biostimulation responds to the market need for organic and more nutritious food [2]. According to the “Global Biostimulants Market 2019-2027”, the global market for biostimulants has been projected to reach $625.67 billion by 2027. The biostimulants improve the nitrogen metabolism, influence the modulation of cell metabolism, control the transfer of nutrients, supervise the lipid biosynthesis, and regulate the stimulation of the soil microbial activity and the root growth [3,4]. Some researchers ascribe their protective effect to the activation of the antioxidant defense system of plants and the production of the phenolic compounds. Low awareness about their efficacy and effects on phytochemical profiles in plants is shown [5,6]. In this study, the effects of Trichoderma harzianum (strains M10 and TH1) or its metabolites, harzianic acid (HA) and 6-pentyl-α-pyrone (6PP), on olive trees (*Olea europaea* var. Ottobriatica) were evaluated. In particular, the antioxidant activity, the phenolic profile, and the concentration of the single phenolic compound on leaf and oil extracts were assessed, since phenolics change the nutritional quality of the oil and influence the interest of the industries in the waste as a source of bioactive components. The effects on these parameters were estimated after drenching and foliar spray applications of the selected biostimulants. Every year, the cultivation of olive fruits produces 25 kilograms of twigs and leaves per tree [7], so recycling them is a priority. These wastes contain bioactive molecules (antioxidants, anti-inflammatory, antitumor, antimicrobial, hypoglycemic, anticholesterol, and antihypertensive agents) [8], which can enhance the economic value of nutraceuticals, functional food, dietary supplements, and nutricosmetic formulations [9].

## 2. Materials and Methods

### 2.1. Plant Material

The impact of bioformulates was tested on *Olea europaea* var. Ottobratica. Ottobratica is an *Olea europaea* variety commonly cultivated in the Calabria Region for oil extraction. The 20-year-old trees situated in the area Rombiolo (Vibo Valentia, South Western Calabria, Italy) were selected and labeled. Plant material used for these experiments was provided by Dr. Andrea Sicari (LINFA scarl, Vibo Valentia, Italy).

Field experiments were chosen with plants (15 years old) in excellent nutritional and phytosanitary status with a low ratio of wood and leaves, and with an adequate number of fruiting branches. The experimental field consisted of 9 rows (3 rows per treatment in a randomized experimental design), each containing 20 plants. Treatments were applied after plants sprouted with a monthly cadence starting from February until July for a total number of 6 treatments. Each bioformulate (10^6^ ufc/mL of the living microbes TH1 (*Trichoderma harzianum*) and M10, 10^−6^ M of the metabolites harzianic acid and 6-pentyl-α-pyrone) and one water treatment (control sample) were applied to the root system by drenching (around the root system at 10 cm deep) and as foliar spray application (10 L per row of which 5 L was drenching and 5 L was spray). The field test was repeated two times. 

### 2.2. Fungal Material

The *Trichoderma* strains were from the microbial collection of the Biological Control laboratories of the University of Naples Federico II. The strains *Trichoderma harzianum* (TH1), *Trichoderma harzianum* (M10 *Trichoderma harzianum* Rifai, anamorph ATCC^®^ 20847™, LGC Standards S.r.l.Sesto, San Giovanni, Italy, and *Trichoderma atroviride strain* P1 (P1) were sustained on PDA medium (potato 125 dextrose agar) (HiMedia, Laboratories Mumbai, India) and protected with sterilized mineral oil (Sigma Aldrich, St. Louis, MO, USA). 

### 2.3. Isolation and Characterization of Harzianic Acid and 6-Pentyl-α-Pyrone

Trichoderma metabolites were isolated and characterized, as reported previously by Pascale et al. [10]. *Trichoderma strains* P1 and M10 were used to arrive at the bioactive compounds. Mycelia were put into 5 L conical flasks containing 1 L of sterile potato dextrose broth (PDB, HiMedia Mumbai, India) for 30 days at 25 °C. A filter paper (Whatman No. 4, Brentford, UK) was used to vacuum-filter the cultures. The filtrate (2 L) was extracted with ethyl acetate (EtOAc). The EtOAc fraction was dried with Na_2_SO_4_ and evaporated in a vacuum at 35 °C. A flash chromatography (50 g Si gel 0.2 e 0.5 mm Merck-EMD Darmstadt, Germany) was used to separate metabolites in the yellow oil residue utilizing a gradient of petroleum ether/EtOAc (9:1 *v*/*v* to 4:6 *v*/*v*) as mobile phase. Then, 80 mg 6PP was obtained. Recovered fractions were chromatographed on thin-layer chromatography (TLC Si gel 60 F_254_ Merck-EMD Darmstadt, Germany; mobile phase, petroleum ether/ethyl acetate, (8:2 *v*/*v*)) and fractions with similar profiles were combined. Successively, the red residue obtained from M10 was dissolved in CHCl_3_, extracted three times with NaOH 2M, and HA precipitated with HCl 2 M. The pellet was recovered (135 mg), solubilized, and subjected to RP-18 vacuum chromatography (20 g Si gel RP-18, 40–63 μm, Sigma Aldrich, St. Louis, MO, USA), eluting with a gradient of CH_3_OH/H_2_O/CH_3_CN (0.5:9:0.5 *v*/*v*/*v* to 10:0:0 *v*/*v*/*v*) to obtain 45 mg of pure HA. The compounds were detected on TLC using UV radiation (254 or 366 nm) and by immersing the sheets in a 5% (*w*/*v*) ethanol solution of 2 M H_2_SO_4_ and heating at 110 °C for 10 min. The purified metabolites were characterized by LC/MS QTOF (Quadrupole Time of Flight) analysis recorded with a 6540 UHD Accurate Mass QTOF LC-MS/MS mass spectrometer (Agilent Technologies, Santa Clara, CA, USA) with a dual ESI (Electrospray Ionization) source, coupled to a 1200 series Rapid Resolution HPLC with a DAD (Diode-Array Detection) system (Agilent Technologies). Sample were identical to standards previously presented at Biological Control laboratories of the University of Naples Federico II.

### 2.4. Chemicals

All the chemicals and standards used were from Sigma Aldrich St. Louis, MO, USA, unless specified differently. Hydroxytyrosol was purchased from Indofine (Hillsborough, NJ, USA) and secologanoside was purchased from ChemFaces Biochemical Co., Ltd. (Wuhan, China).

### 2.5. Analytical Methods

#### 2.5.1. Ultra High-Performance Liquid Chromatograph

Qualitative and quantitative profiles of phenols were obtained using an Ultra-High-Performance Liquid Chromatography (UHPLC, Thermo Fisher Scientific, Waltham, MA, USA) provided with a degassing system (Dionex Ultimate 3000), a quaternary UHPLC pump working at 1250 bar, an autosampler device, and a thermostated column compartment (T = 30 °C) with a Accucore aQ 2.6 µm (100 × 2.1 mm) column (Thermo Scientific, Waltham, MA, USA). The injection volume was 5 µL. The mobile phase was made as follows: Phase A: H_2_O 0.1% of acetic acid, and phase B: Acetonitrile. Phenols were eluted using a 0.4 mL/min flow rate with a gradient programmed: 0 to 5 min 5% phase B, 6 to 25 min 40% phase B, 25.1 to 27 min 100% phase B, 27.1 to 35 min 5% phase B, 35.1 to 45 min 0% phase B. Phenolics’ identification occurred by comparing the retention times to the mass spectra of the purified compounds and the standards. The ligstroside quantification was made using the oleuropein in place of the authentic standard not commercially available.

#### 2.5.2. Mass Spectrometry Analysis

Mass experiments were done with a Q Exactive Orbitrap LC-MS/MS (Thermo Fisher Scientific, Waltham, MA, USA) equipped with an ESI source (HESI II, Thermo Fisher Scientific, Waltham, MA, USA) operating in negative ion mode (ESI−). The ion source parameters were spray voltage −3.0 kV, sheath gas (N_2_ > 95%) 30, auxiliary gas (N_2_ > 95%) 15, capillary temperature 200 °C, S-lens RF level 50, auxiliary gas heater temperature 305 °C. The MS (Mass) detection was conducted in two acquisition modes: Full scan (negative-ion modes) and targeted selected ion monitoring. The parameters of the full scan acquisition mode were: Mass resolving power 35,000 full width at half maximum (at *m*/*z* 200), scan range 100–1500 *m*/*z*, scan rate 2s^−1^, and the automatic gain control target 1 × 10^5^ ions for a maximum injection time of 200 ms. The parameters of the targeted selected ion monitoring acquisition mode were: 15s-time window, resolution power 35,000 full width at half maximum (at *m*/*z* 200), and quadrupole isolation window 1.2 *m*/*z*. The mass list containing exact masses and expected retention times of the target polyphenolic compounds was taken in. 

#### 2.5.3. Q Exactive Orbitrap UHPLC-MS/MS Method Validation

The method linearity was evaluated by the regression coefficient of the calibration curve. The calibration curve was determined by using phenolics external standards. Limits of detection (LODs) and limits of quantification (LOQs) were derived from the regression curve (LODs = 3 × $\;\frac{\mathrm{standard}\;\mathrm{deviation}}{angular\;coefficient}$; LOQs = 10 × $\frac{\mathrm{standard}\;\mathrm{deviation}}{angular\;coefficient}$). Intraday repeatability was evaluated by injecting seven different concentrations of each phenolic standard three times. The interday assay variations were obtained by repeating after seven days the same operating conditions.

#### 2.5.4. Extraction of Phenolics from the Olive Oil 

The phenolic compounds were extracted following the method of Vasquez Roncero [11]. In brief: 25 g of oil were dissolved in 25 mL hexane. The polar compounds were extracted three times, with 15 mL of a mixture consisting of methanol:water (3:2 *v*/*v*). The three extracts were mixed and treated once with 25 mL hexane. The solvent was evaporated to dryness in a rotary evaporator (Buchi, Switzerland) at 40 °C. The residue was dissolved in 1 mL of methanol and filtered through 0.2 mm nylon filer and immediately frozen and stored at −18 °C until analysis.

#### 2.5.5. Extraction of Phenolics from the Olive Leaves

The sample extraction was performed as described previously by Talhaoui et al. [12]. The dry leaves (0.5 g) were crushed and extracted with MeOH/H_2_O (80:20 *v*/*v*) (10 mL). The samples were placed in an ultrasonic bath for 10 min and then centrifuged at 6000 rpm for 10 min. The supernatant was recovered, and the pellet was re-extracted following the procedure applied to the starting sample. The obtained supernatants were dried to rotavapor and dissolved in 4 mL of a methanol/water solution (50:50 *v*/*v*). Three replicates of each sample were processed. 

#### 2.5.6. Estimation of the Antioxidant Activity

The 2.2-diphenyl-1-picrylhydrazyl (DPPH) radical-scavenging capacity was assessed utilizing the method described by Brand-Williams et al. (1995) [13]. The phenolic extract (20 μL) was added to 3 mL of DPPH solution (6 × 10^−5^ mol/L), and the absorbance was determined at 517 nm every 5 mins until the steady state (Lambda 25, PerkinElmer, Italy). 

#### 2.5.7. Statistical Analysis

All analyses were made with “Statistica” software version 7.0 (StatSoft, Inc, Tulsa, OK, USA). 

## 3. Results

The hydrophilic extracts obtained from EVOO and olive leaves were analyzed by Q Exactive Orbitrap UHPLC-MS/MS to identify and quantify the main phenolics. 

### 3.1. Validation of the MS Method 

Validation parameters for the analysis of the phenolic by UPLC-HRMS-Orbitrap are reported in Table 1.

### 3.2. Identification of the Phenolic Compounds

Nineteen phenolics, including two flavonoids, two phenolic alcohols, six phenolic acids, two lignans, and seven secoiridoids, were identified and quantified. The parameters used to identify phenolics in samples are reported in Table 2.

### 3.3. Quantification of the Phenolic Compound

Different amounts of single phenolics were quantified (Table 3 Table 4 Table 5 Table 6). In vivo experiment for the disease control of the olive plants showed that not only the *Trichoderma* strains but also their metabolites (6PP and HA) in all samples tested had a positive effect on the luteolin, the pinoresinol, and the acetoxypinoresinol content (Table 3).

The biostimulation decreased the tyrosol concentration in all samples tested and enhanced the hydroxytyrosol concentration in the EVOO samples (Table 4).

The response of the phenolic acids to the treatments was very different in the EVOO and the leaf samples. All biostimulators had a positive effect in the leaf samples on the 4-hydroxybenzoic acid, the vanillic acid, and the cinnamic acid content, and a positive impact in the EVOO samples on the 3-hydroxybenzoic acid, the vanillic acid, the p-coumaric acid and the ferulic acid content (Table 5).

Finally, all biostimulant treatments positively affected oleuropein content (Table 6).

### 3.4. Antioxidant Activity 

**Figure 1:**
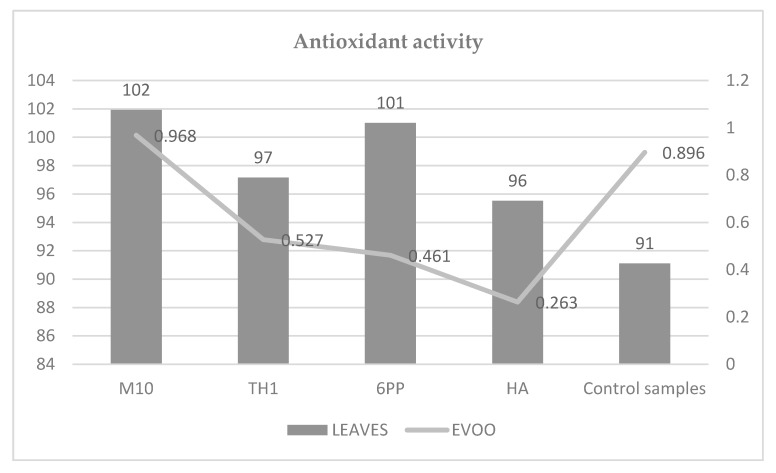
Antioxidant activities by analyzing samples (mmol Trolox/kg).

The biostimulation of olive trees had a positive effect on the antioxidant activity of the olive leaf samples and of the EVOO samples obtained from the olive tree under M10 treatment (Figure 1). 

## 4. Discussion

**Figure 2:**
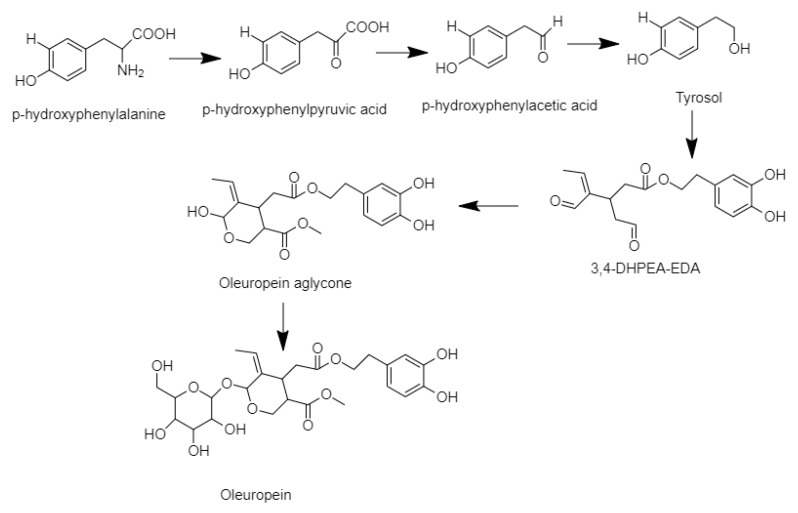
Oleuropein biosynthesis [33].

Olive oil and nonfood olive biomass are promising sources of bioactive molecules, including phenols. The latter, when added to food, cosmetics, and medicines have additive (flavoring, coloring, and texturizing) and functional properties [8,14,15,16,17]. The plants produce phenolics, mainly in leaves or bark, to attract pollinators, protect against UV radiation, and defend against phytopathogenic bacteria, viruses, and fungi [18]. In this study, the phenolic profile and dosage in extra-virgin olive oil and olive leaves were investigated by using an HPLC-Orbitrap platform in MS and MS/MS levels. The linearity, sensitivity, and precision were studied to validate the MS methods. The correlation factor of the calibration curve = 0.999 and the residues normally distributed confirmed the linearity of the calibration curve, the method sensitivity in the range of the LODs and the LOQs, verified method sensitivity, and the RSD (Relative Standard Deviation) values <15% validated the inter- and intraday repeatability. Two flavonoids, two lignans, seven secoiridoids, six phenolic acids, and two phenolic alcohols were identified and quantified. Identification was made comparing the retention time, mass spectra, accurate mass measurements, and MS2 analyses with standards when they were commercially available or with literature data (ligstroside) [19]. The presence of two hydroxybenzoic acid isomers was confirmed comparing the retention time and mass spectra with standards. The flavonoids and the lignans occurred in the aglycon form in the oil and the leaf samples. It was probably due to degradation during the malaxation process and leaf breakage to allow extraction. The biostimulation treatment increased the concentrations of flavonoids and the lignans in the EVOO and the leaves, although organic agriculture generally affects negatively on polyphenol contents, since the shikimate pathway links the biosynthesis of these compounds and nitrogen metabolism. The protein-phenol competition decreased the action of the phenylalanine ammonia-lyase, an enzyme involved in their biosynthesis [20,21,22]. Instead, *Trichoderma* strains may improve phenolic concentrations, increasing the plant nutrient uptake mechanism [23] and enhancing plant nitrogen use efficiency [24,25]. Our results support that not only Trichoderma strains have this ability, but, also, their metabolites (6PP and HA) increase the concentration of the polyphenols both in olive leaves and in oil. These findings revealed that the 6PP and the HA are involved in the induction of plant systemic resistance. The different concentrations of the polyphenols under the microbe or the microbe metabolites biostimulation were related to the ability of the living microbe to attach the pathogen by more than one mechanism, including the antibiosis, the competition, the direct mycoparasitism, and the induction of the disease resistance that acts synergistically [24]. The higher concentration of flavonoids in the leaves than in the oil can be explained since the flavonoids are produced in the leaves [26]. The prevalence of the luteolin, among the flavonoids, agreed with the results already detected for the olive species [27]. The high content of luteolin was of great interest, as its intake was associated with antioxidant and health-promoting actions [28,29]. Other phenolics synthesized through the shikimic pathway are phenolic acids [30]. In this study, all biostimulation treatments had a positive effect only on vanillic acid concentration. The concentration of the phenolic acids was affected by the ripening process, except for the vanillic acid content [31]. Therefore, the vanillic acid content was only affected by the effects due to the use of biostimulants in agriculture. Concerning secoiridoid fractions, the biostimulation decreased oleuropein degradation. The higher concentration of oleuropein and the lower concentration of its degradation product (tyrosol) demonstrated it [32] (Figure 2). 

The oleuropein has nutraceutical and pharmaceutical properties. It modulates several oncogenic signaling pathways and treats oxidant and inflammatory-related diseases (obesity, diabetes, neuro deficiency, cardiovascular disease, and hepatic disorder) [34]. Finally, the antioxidant activity was evaluated. In the leaf tests, all biostimulation treatment increased the antioxidant potential of the samples. On the reverse, in EVOO experiments, only the M10 stimulation gave these answers. In this investigation, antioxidant activity was founded by the DPPH test. This method limited the capability of the antioxidant to transfer an electron and to reduce carbonyls, metals, and themes. It provides the highest rankings to the compound with many phenol groups and ortho-substituted with electron-donating alkyl or methoxy groups [35,36]. In EVOO samples, the most representative phenolics (tyrosol, hydroxytyrosol, oleacein, oleuropein-aglycone mono-aldehyde, and ligstroside aglycone-dialdehyde) did not have these characteristics. Thus, the antioxidant potential of the EVOO samples may have been underestimated [37]. On the contrary, in the leaf samples, the B-ring catechol structure and the 2.3-double bond conjugated with a 4-oxo in the structure of the most representative compound (luteolin) may have resulted in an overestimation of the antioxidant potential.

## 5. Conclusions

In this study, the effects of olive trees’ treatment with beneficial microbes belonging to *Trichoderma* genera were evaluated in extra-virgin olive oil and olive leaves on phenolic profile and content by using HPLC-Orbitrap platform in MS and MS/MS levels. The recovery, the linearity, the precision, and the accuracy validated the used methods. This study supported the excellent effect of biostimulation on the nutraceutical value of EVOO, and for the first time delineated the effects of the Trichoderma secondary metabolites (6PP and the HA) used in the olive tree cultivation on the phenolics’ production and the antioxidant activity in EVOO and leaf samples. Our results suggest the possibility of using HA and 6PP in olive cultivation, as they eliminate some of the limitations related to the application of living biological control agents and the concentration of phenolic compounds in olive leaves, and making these wastes commercially attractive as a source of phytochemicals useful for the pharmaceutical, cosmetic, and food industries.

## Figures and Tables

**Table 1:** Validation parameters for phenolics analysis by UPLC-HRMS-Orbitrap.

Phenolic Compounds	Linearity (mg/L)	R^2^	LOD (mg/L)	LOQ (mg/L)	Intraday RSD % (*n* = 3), 50 mg/L
Phenolic Acids					
					
4-Hydroxybenzoic acid	1–50	0.998	0.207	0.622	0.9
3-Hydroxybenzoic acid	1–50	0.995	0.205	0.622	1.1
Vanillic acid	1–50	0.887	0.200	0.600	1.1
Cinnamic acid	1–50	0.991	0.200	0.600	0.9
*p*-Coumaric acid	1–50	1.000	0.100	0.300	1.8
Ferulic acid	1–50	0.912	0.100	0.300	1.7
**Flavonoids**					
Luteolin	0.5–50	0.991	0.066	0.200	1.4
Apigenin	0.5–50	0.899	0.066	0.200	2.1
**Lignans**					
(+)Pinoresinol	1–50	0.999	0.02	0.060	0.5
(+)1-Acetoxypinoresinol	1–50	0.899	0.233	0.700	1.5
**Phenolic Alcohols**					
*p*-HPEA (Tyrosol)	1–50	0.991	0.133	0.040	1.6
3.4 DHPEA (Hydroxytyrosol)	1–50	0.992	0.666	2.000	3.0
**Secoiridoids**					
Oleuropein	1–50	0.991	0.166	0.500	5.0
Ligstroside	1–50	0.991	0.166	0.500	4.0
Secologanoside	1–50	0.967	0.333	1.000	2.1
Elenaic acid	1–50	0.991	0.333	1.000	0.7
3.4 DHPEA-EDA (Oleuropein aglycone dialdehyde) 3.4-DHPEA-EA (Oleuropein-aglycone monoaldehyde)	1–50	0.998	1.000	3.000	2.1
*p*-HPEA-EDA (Ligstroside-aglycone dialdehyde)	1–50	0.899	0.416	1.250	3.0

**Table 2:** Phenolics’ identification parameters.

Phenolic Compounds	RT (min)	Formula	Theoretical *m*/*z* of deprotonated molecular ions [M − H]^−^	Experimental *m*/*z* of deprotonated molecular ions [M − H]^−^	Calculated Errors ∆ppm	Fragments	Collision Energy (eV)
Phenolic Acids							
4-Hydroxybenzoic acid	2.57	C_7_H_6_O_3_	137.02442	137.02456	1.02	93.03431	12
3-Hydroxybenzoic acid	2.88	C_7_H_6_O_3_	137.02442	137.02458	1.17	93.03431	12
Vanillic acid	4.30	C_8_H_8_O_4_	167.03498	167.03522	1.44	152.01143	20
Cinnamic acid	11.54	C_9_H_8_O_2_	147.04515	147.04536	1.43	103.04501	20
*p*-Coumaric acid	9.71	C_9_H_10_O_5_	163.04007	163.04028	1.29	119.05023	20
Ferulic acid	11.81	C_10_H_10_O_4_	193.05063	193.05084	1.09	178.02685	20
**Flavonoids**							
Luteolin	19.07	C_15_H_10_O_6_	285.04046	285.04106	2.10	133.02940	30
Apigenin	19.12	C_15_H_10_O_5_	269.04555	269.04597	1.56	225.05592	35
**Lignans**							
(+) Pinoresinol	17.00	C_20_H_22_O_6_	357.13436	357.13487	1.43	151.03961	40
(+) 1-Acetoxypinoresinol	19.10	C_22_H_24_O_8_	415.13984	415.14007	0.55	415.13821	40
**Phenolic Alcohols**							
Tyrosol (*p*-HPEA)	2.75	C_8_H_10_O_2_	137.06080	137.06096	1.17	119.05022	12
Hydroxytyrosol (3,4 DHPEA)	1.60	C_8_H_10_O_3_	153.05572	153.05580	0.52	123.04561	12
**Secoiridoids**							
Oleuropein	16.69	C_25_H_32_O_13_	539.17701	539.17767	1.22	377.12393	20
Ligstroside	18.25	C_25_H_32_O_12_	523.18210	523.18279	1.32	361.12914	12
Secologanoside	19.49	C_16_H_21_O_11_	389.1092	389.109258	0.59	345.1195	12
Elenaic acid	13.14	C_11_H_14_O_6_	241.07176	241.07212	1.49	209.04573	10
Oleacein (3.4 DHPEA-EDA)	16.14	C_17_H_20_O_6_	319.11871	319.11898	0.85	301.1082	15
Oleuropein-aglycone mono-aldehyde (3.4 DHPEA-EA)	21.25	C_19_H_22_O_8_	377.12419	377.12442	0.61	345.09790	12
Ligstroside-aglycone dialdehyde (*p*-HPEA-EDA)	18.59	C_17_H_20_O_5_	303.12380	303.12441	2.01	301.1082	12

**Table 3:** Flavonoids and lignans in leaf and extra-virgin olive oil (EVOO) samples.

Flavonoids (mg/kg)			Lignans (mg/kg)	
	Luteolin	Apigenin	Pinoresinol	Acetoxypinoresinol
**LEAF Samples**			**LEAF Samples**	
M10	5376.796 ± 19.561 (+23%)	386.225 ± 2.12 (+1%)	29.932 ± 0.038 (+56%)	151.353 ± 3.269 (+58%)
TH1	5609.046 ± 56.141 (+26%)	349.017 ± 8.094 (−10%)	26.813 ± 2.045 (+50%)	176.758 ± 6.716 (+64%)
6PP	10163.237 ± 50.790 (+59%)	540.855 ± 3.289 (+29%)	33.476 ± 2.813 (+60%)	161.364 ± 11.729 (+61%)
HA	8728.101±185.859 (+53%)	627.880 ± 0.879 (+ 39%)	35.846 ± 1.352 (+63%)	126.704 ± 3.764 (+50%)
Control	4134.259 ± 47.604	382.677 ± 0.560	13.278 ± 0.157	63.529 ± 0.290
**EVOO Samples**			**EVOO Samples**	
M10	7.317 ± 0.054 (+57%)	0.251 ± 0.005 (+10%)	0.203 ± 0.013 (+53%)	9.829 ± 0.035 (+56%)
TH1	7.648 ± 0.072 (+58%)	0.182 ± 0.002 (−26%)	0.22 ± 0.009 (+57%)	7.743 ± 0.062 (+44%)
6PP	6.145 ± 0.009 (+48%)	0.201 ± 0.001 (−13%)	0.172 ± 0.004 (+45%)	11.52 ± 0.308 (+62%)
HA	10.218 ± 0.014 (+70%)	0.221 ± 0.001 (−3%)	0.152 ± 0.005 (+37%)	8.355 ± 0.14 (+48%)
Control	3.178 ± 0.046	0.228 ± 0.001	0.095 ± 0.007	4.344 ± 0.097

% improvement of samples compared to control.

**Table 4:** Phenolic alcohol compounds in the leaf and the EVOO samples.

Phenolic Alcohols (mg/kg)		
	Tyrosol	Hydroxytyrosol
**LEAF Samples**		
M10	12.897 ± 1.325 (−390%)	0.928 ± 0.008 (+31%)
TH1	11.524 ± 0.034 (−448%)	0.498 ± 0.016 (−28%)
6PP	12.428 ± 0.269 (−408%)	0.535 ± 0.01 (−19%)
HA	12.198 ± 1.342 (−418%)	0.683 ± 0.005 (+7%)
Control	63.149 ± 2.143	0.636 ± 0.007
**EVOO Samples**		
M10	105.917 ± 1.698 (−325%)	595.136 ± 17.946 (+74%)
TH1	153.018 ± 0.253 (−194%)	236.603 ± 6.405 (+35%)
6PP	156.413 ± 1.237 (−188%)	504.858 ± 3.7 (+70%)
HA	118.196 ± 0.443 (−281%)	180.862 ± 3.789 (+15%)
Control	450.646 ± 6.736	152.706 ± 0.424

% improvement of samples compared to control.

**Table 5:** Phenolic acids in the leaf and the EVOO samples.

Phenolic Acid (mg/kg)						
	4-Hydroxybenzoic Acid	3-Hydroxybenzoic Acid	Vanillic Acid	*p*-Coumaric Acid	Cinnamic Acid	Ferulic Acid
**LEAF Samples**						
M10	27.29 ± 1.787 (+42%)	229.718 ± 2.840 (−7%)	159.895 ± 0.498 (+41%)	27.859 ± 0.092 (−116%)	3.954 ± 0.06 (+67%)	21.646 ± 0.023 (−70%)
TH1	20.41 ± 0.677 (+22%)	235.590 ± 8.660 (−4%)	168.819 ± 6.591 (+44%)	23.787 ± 1.112 (−152%)	2.986 ± 0.008 (+57%)	22.456 ± 0.916 (−64%)
6PP	19.908 ± 0.227 (+20%)	256.863 ± 0.801 (−4%)	182.615 ± 3.141 (+48%)	43.651± 0.342 (−38%)	1.150 ± 0.061 (−12%)	30.275 ± 0.107 (−22%)
HA	20.190 ± 0.398 (+21%)	249.687 ± 2.176 (+1%)	178.680 ± 4.492 (+47%)	26.627 ± 0.434 (−125%)	2.651±0.33 (+51%)	23.313 ± 0.279 (−58%)
Control	15.833 ± 0.102	245.931±1.295	94.195 ± 0.497	60.067 + 0.865	1.287 + 0.031	36.836 ± 0.093
**EVOO Samples**						
M10	0.883 ± 0.007 (+31%)	0.796 ± 0.004 (+66%)	7.05 ± 0.059 (+62%)	3.274 ± 0.024 (+56%)	0.482 ± 0.009 (+9%)	0.131 ± 0.001 (+51%)
TH1	0.473 ± 0.015 (−28%)	0.845 ± 0.005 (+68%)	8.35 ± 40.006 (+68%)	3.422 ± 0.032 (+58%)	0.348 ± 0.003 (−26%)	0.219 ± 0.005 (+71%)
6PP	0.509 ± 0.009 (−27%)	0.75 ± 0.005 (+64%)	7.814 ± 0.067 (+66%)	2.749 ± 0.004 (+48%)	0.386 ± 0.002 (−13%)	0.125 ± 0.003 (+49%)
HA	0.649 ± 0.005 (+7%)	1.172 ± 0.012 (+77%)	13.17 ± 0.116 (+80%)	4.572 ± 0.006 (+69%)	0.423 ± 0.002 (−3%)	0.238 ± 0.000 (+73%)
Control	0.605 ± 0.007	0.27 ± 0.003	2.663 ± 0.012	1.422 ± 0.021	0.438 ± 0.002	0.064 ± 0.000

% improvement of samples compared to control.

**Table 6:** Secoiridoids in the leaf and the EVOO samples.

Secoiridoid (mg/kg)							
	Ligstroside	Oleuropein	Secologanoside	Elenaic Acid	Oleacein	Oleuropein-Aglycone Mono-Aldehyde	Ligstroside-Aglycone di-Aldehyde
**LEAF Samples**							
M10	907.225 ± 25.038 (+69%)	15225.842 ± 261.018 (+41%)	307.740 ± 9.109 (+3%)	370.346 ± 6.552 (−105%)	6442.141 ± 43.543 (+87%)	344.531 ± 5.578 (−71%)	117.220 ± 2.866 (−34%)
TH1	754.591 ± 12.805 (+62%)	14230.720 ± 297.140 (+36%)	252.887 ± 9.855 (−18%)	568.815 ± 6.404 (−33%)	273.507 ± 2.253 (−209%)	178.034 ± 4.785 (−230%)	24.547 ± 0.547 (−547%)
6PP	238.574 ± 4.964 (−19%)	11784.606 ± 108.787 (+23%)	274.257 ± 3.357 (−8%)	561.516 ± 2.589 (−35%)	2650.549 ± 2.611 (+68%)	247.182 ± 0.604 (−138%)	152.650 ± 0.571 (−3%)
HA	217.621 ± 7.405 (−31%)	10272.050 ± 223.885 (+12%)	274.860 ± 7.500 (−8%)	498.886 ± 7.152 (−52%)	364.933 ± 6.272 (−131%)	124.221 ± 2.778 (−373%)	55.490 ± 0.108 (−194%)
Control	284.823 ± 2.205	9043.638 ± 189.420	297.833 ± 1.635	758.908 ± 22.919	844.611 ± 5.676	587.819 ± 5.041	157.254 ± 1.435
**EVOO Samples**							
M10	0.009 ± 0.001 (−433%)	0.145 ± 0.029 (+4%)	0.391 ± 0.002 (+52%)	15.494 ± 0.13 (+62%)	1031.14 ± 9.208 (−8%)	344.531 ± 5.578 (−71%)	712.316 ± 0.683 (−42%)
TH1	0.069 ± 0.01 (+30%)	0.152 ± 0.01 (+8%)	0.205 ± 0.003 (+10%)	18.36 ± 0.014 (+68%)	871.855 ± 0.506 (−27%)	178.034 ± 4.785 (−230%)	519.144 ± 3.714 (−95%)
6PP	0.007 ± 0.002 (−586%)	0.15 ± 0.00 (+7%)	0.207 ± 0.006 (+10%)	17.174 ± 0.11 (+66%)	736.801 ± 8.596 (−491%)	247.182 ± 0.604 (−138%)	505.106 ± 4.879 (−100%)
HA	0.008 ± 0.002 (−500%)	0.155 ± 0.013 (+10%)	0.205 ± 0.00 (+10%)	28.945 ± 0.255 (+80%)	481.884 ± 0.819 (−128%)	124.221 ± 2.778 (−373%)	379.34 ± 2.044 (−166%)
Control	0.048 ± 0.007	0.139 ± 0.007	0.186 ± 0.01	5.854 ± 0.026	1100.931 ± 5.181	587.819 ± 5.041	1011.245 ± 7.233

% improvement of samples compared to control.
